# Undifferentiated Pleomorphic Sarcoma Mimicking a Refractory Thigh Abscess in a Young Adult: A Diagnostic Pitfall

**DOI:** 10.7759/cureus.110666

**Published:** 2026-06-11

**Authors:** Abisiniya Kabtimer, Grygoriy Gavrylyuk, Matthew Furman

**Affiliations:** 1 Surgery, BronxCare Health System, Bronx, USA

**Keywords:** fnclcc grade 3, mdm2 negative, misdiagnosis, soft tissue sarcoma, thigh mass, undifferentiated pleomorphic sarcoma, wide local excision, young adult

## Abstract

Undifferentiated pleomorphic sarcoma (UPS) is a high-grade sarcoma typically seen in older adults; its occurrence in young adults is rare and poses significant diagnostic challenges. We report the case of a 21-year-old female who initially presented with a presumed infectious lesion that proved refractory to standard antibiotic therapy and incision and drainage. Progressive enlargement prompted surgical excision, with pathology demonstrating FNCLCC grade 3 UPS. Histopathologic evaluation revealed a pleomorphic high-grade neoplasm with extensive necrosis and high mitotic activity, while immunohistochemical and molecular studies, including negative MDM2 amplification, supported the diagnosis of UPS. Staging studies demonstrated no evidence of metastatic disease. The patient subsequently underwent wide local re-excision with negative margins, and postoperative MRI demonstrated no residual enhancing mass. During subsequent follow-up, the patient continued oncologic care at Memorial Sloan Kettering Cancer Center. Documentation from December 2025 reported multiple pulmonary lesions identified on surveillance CT imaging. Subsequent biopsy confirmed metastatic sarcoma involving the lungs. The patient was subsequently enrolled in an immunotherapy-based clinical trial at the outside institution. The patient also continued routine follow-up at our institution every six months. Local examination of the right posterior thigh surgical site demonstrated no evidence of local recurrence. At the most recent documented follow-up in March 2026, the patient remained under active multidisciplinary oncologic management with ongoing surveillance. This case highlights the importance of considering soft tissue sarcoma in atypical or nonresolving soft tissue lesions to avoid delays in diagnosis and management.

## Introduction

Undifferentiated pleomorphic sarcoma (UPS), formerly termed malignant fibrous histiocytoma, was first described by O’Brien et al. in 1964 and is now classified as a high-grade soft tissue sarcoma of uncertain histogenesis. It is composed of markedly pleomorphic spindle and polygonal cells arranged in a storiform pattern, often accompanied by multinucleated giant cells and inflammatory elements. UPS most commonly arises in the extremities, particularly the lower limbs, and predominantly affects older adults, with a peak incidence in the sixth decade and a slight male predominance [1,2]. Although rare in younger populations, UPS has been reported in adolescents and young adults, accounting for approximately 3-5% of cases [1]. In this age group, its clinical behavior, molecular profile, and outcomes may differ from those observed in older patients, further complicating diagnosis and management [1,3].

Clinically, UPS most often presents as a painless, progressively enlarging soft tissue mass. However, atypical presentations can occur and may mimic benign or infectious conditions, leading to delays in diagnosis and treatment [4,5]. Imaging and histopathologic evaluation, including immunohistochemistry and molecular testing such as MDM2 amplification analysis, are essential to establish the diagnosis and exclude histologic mimics such as dedifferentiated liposarcoma [6]. UPS is a high-grade malignant mesenchymal neoplasm characterized by marked cellular atypia and the absence of an identifiable line of differentiation, making it a diagnosis of exclusion. Complete surgical excision with negative margins remains the mainstay of treatment [6,7].

Here, we present a case of high-grade UPS arising in the posterior thigh of a 21-year-old female, initially misdiagnosed as an infectious lesion. This case highlights the diagnostic challenges associated with atypical presentations in young patients and underscores the importance of early suspicion and appropriate oncologic management.

## Case presentation

A 21-year-old female with no significant past medical or surgical history presented with a progressively enlarging lesion on the right posterior thigh, first noted in April 2025. Family history was notable for breast cancer in her mother, diagnosed in 2023.

**Figure 1:**
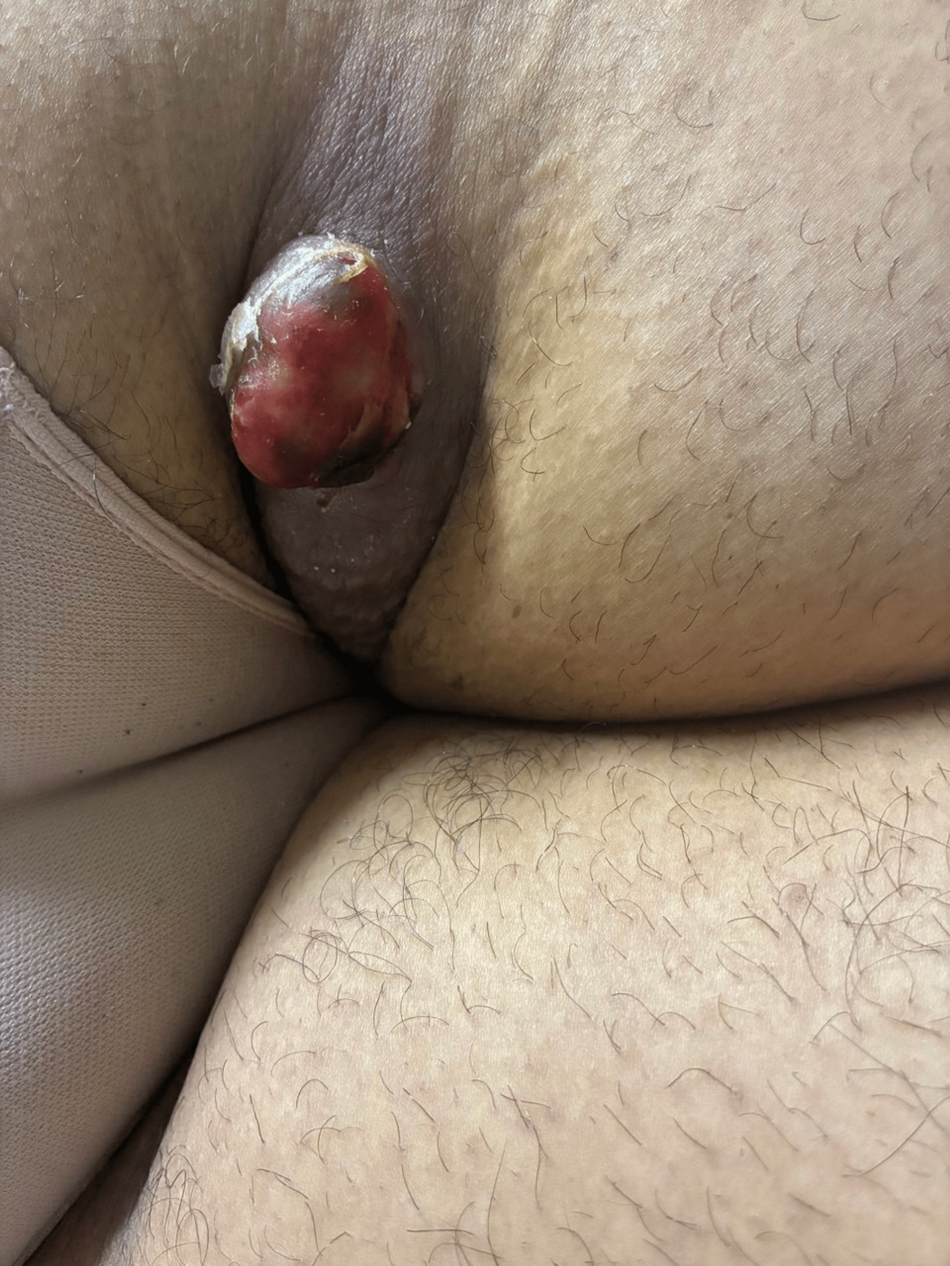
Initial clinical presentation of the right posterior thigh lesion Clinical photograph demonstrating an erythematous, fluctuant soft tissue lesion initially presumed to represent a furuncle or abscess prior to definitive diagnosis.

On August 13, 2025, she was evaluated by her primary care physician. Physical examination revealed a 4 × 2.5 cm lesion described as a “furuncle” with central fluctuance. She was prescribed amoxicillin-clavulanate. On August 22, 2025, she presented to the emergency department with worsening symptoms despite antibiotic therapy. Examination demonstrated erythema and a small fluctuant area (Figure 1), and the lesion was treated as an abscess. Incision and drainage were performed at an outside facility, yielding a small amount of blood-tinged purulent material, and she was discharged on cephalexin and trimethoprim-sulfamethoxazole. The patient reported only a mild decrease in the size of the lesion following the procedure, without sustained improvement, and the mass continued to enlarge thereafter.

**Figure 2:**
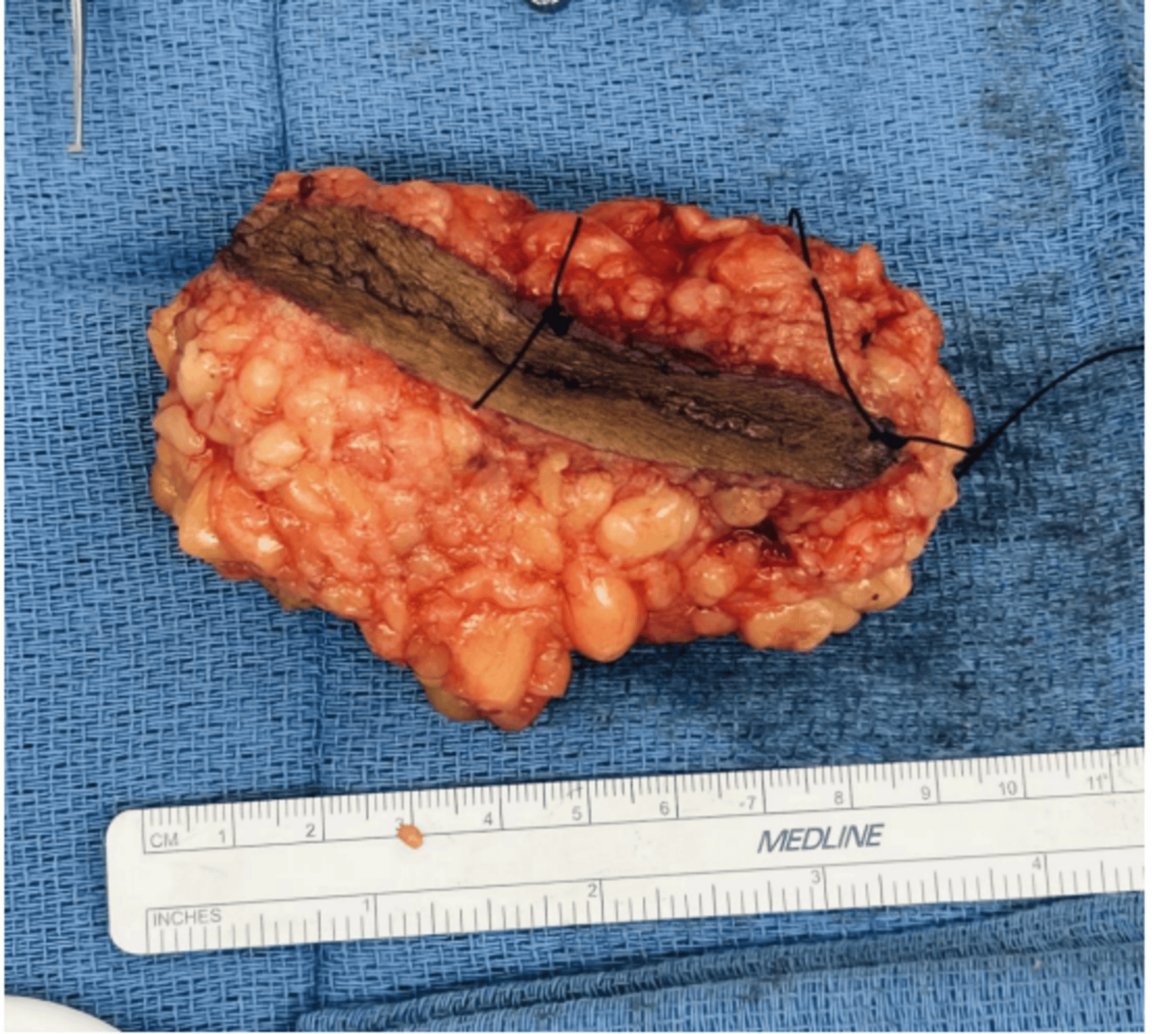
Gross appearance of the excised right posterior thigh mass following initial surgical resection The specimen measured approximately 8 cm in greatest dimension, as demonstrated by the adjacent ruler.

**Figure 3:**
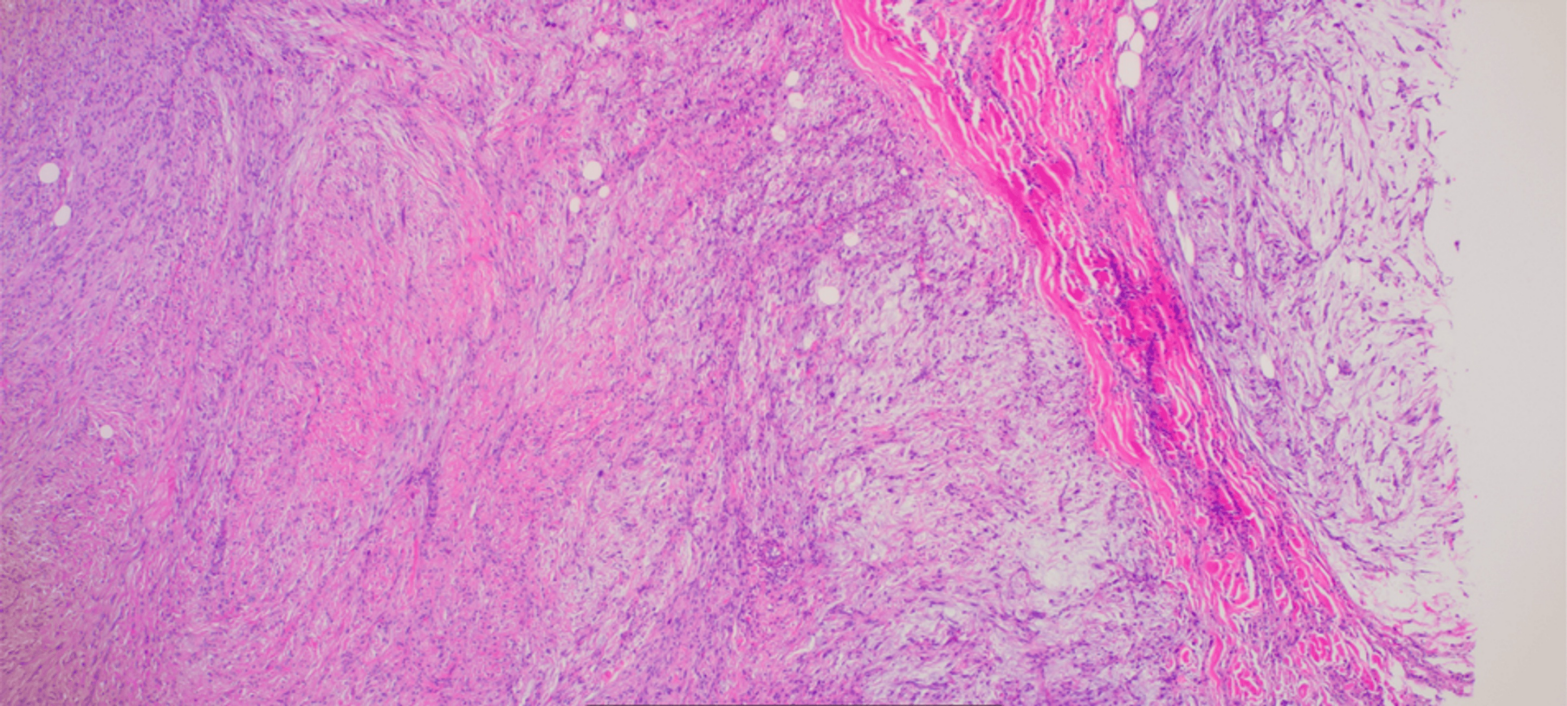
Low-power histopathologic view of UPS Low-power H&E-stained section of the original right posterior thigh tumor demonstrating a heterogeneous neoplasm with mixed solid fibrotic areas and less cellular myxoid regions, consistent with UPS. UPS, undifferentiated pleomorphic sarcoma

**Figure 4:**
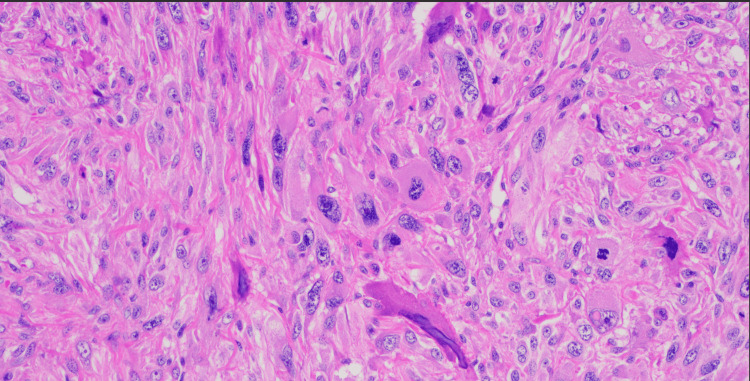
High-power histopathologic view of UPS High-power (40×) H&E-stained section of the original right posterior thigh tumor demonstrating markedly pleomorphic tumor cells with enlarged atypical nuclei, abundant eosinophilic cytoplasm, and frequent atypical mitotic figures, consistent with high-grade UPS. UPS, undifferentiated pleomorphic sarcoma

**Figure 5:**
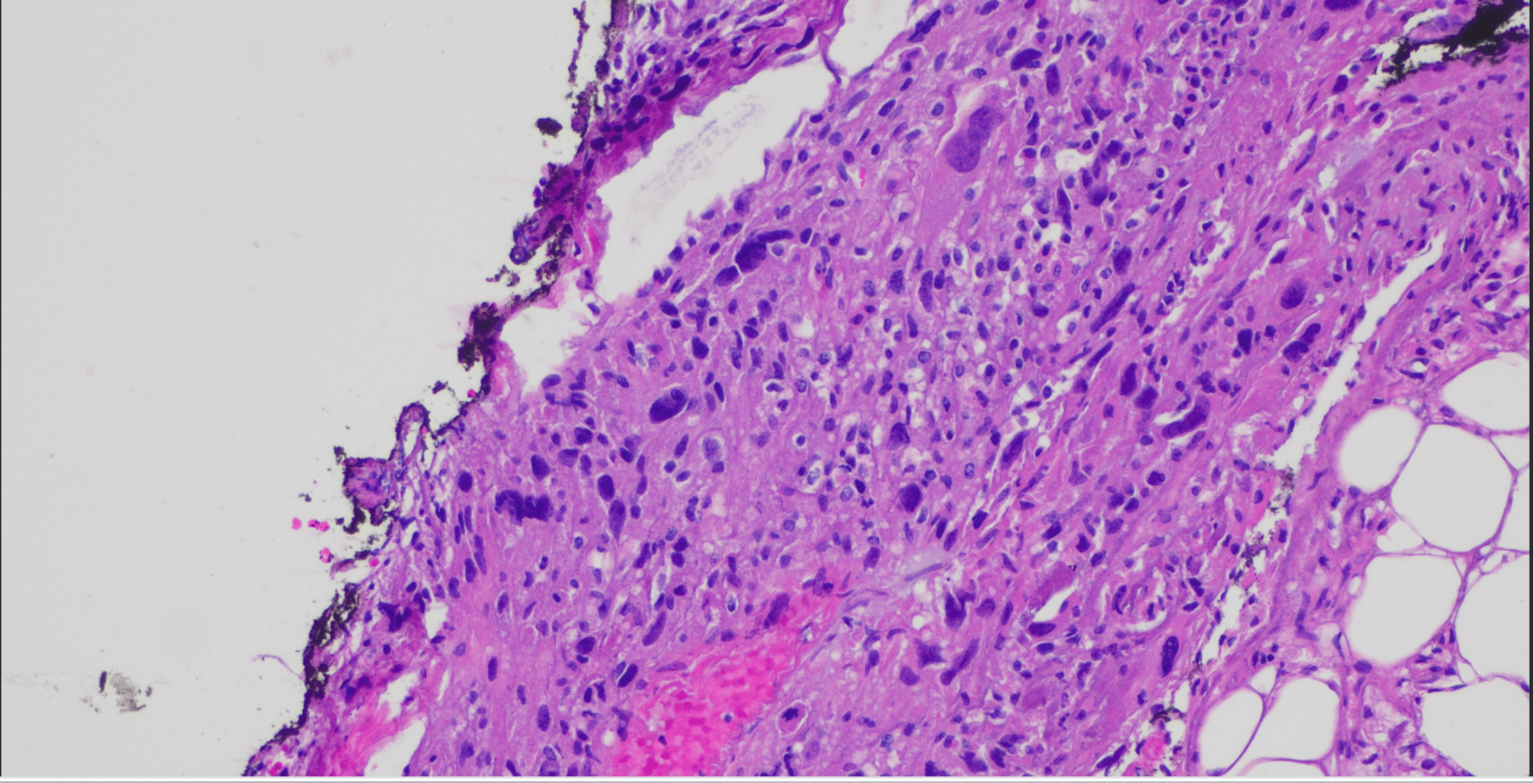
Tumor involvement of the surgical margin in UPS H&E-stained section of the initial resection specimen demonstrating tumor involvement of the inked surgical margin, confirming incomplete excision of the UPS. UPS, undifferentiated pleomorphic sarcoma

**Figure 6:**
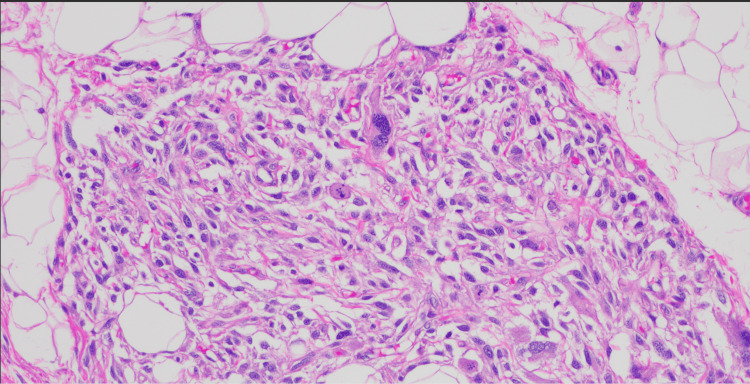
Histopathologic features of residual tumor in re-excision specimen Re-excision specimen obtained on November 20, 2025 showing a high-grade pleomorphic neoplasm with histologic features similar to the original tumor, consistent with undifferentiated pleomorphic sarcoma. UPS, undifferentiated pleomorphic sarcoma

Despite these interventions, the lesion continued to enlarge. On September 12, 2025, surgical evaluation revealed a 6 × 3 × 2 cm mass. Imaging suggested an exophytic lesion, prompting referral to surgical oncology. On September 18, 2025, the patient underwent excision of the mass. Intraoperatively, an 8 cm lesion was identified and removed (Figure 2). Histopathological examination demonstrated a high-grade pleomorphic neoplasm consistent with UPS, FNCLCC grade 3. Low-power imaging (Figure 3) demonstrated mixed fibrotic and myxoid tumor architecture, while high-power imaging (Figure 4) showed marked cellular pleomorphism and atypical mitotic figures. Margin assessment (Figure 5) confirmed tumor involvement of the inked surgical margin. The re-excision specimen (Figure 6) demonstrated a tumor with histologic features similar to the original lesion, consistent with persistent undifferentiated pleomorphic sarcoma. MDM2 and CDK4 amplification testing was negative.

Table 1 summarizes the patient’s clinical course from initial symptom onset through diagnosis, surgical management, metastatic progression, and most recent follow-up.

**Table 1:** Clinical timeline of presentation, diagnosis, treatment, and follow-up UPS, undifferentiated pleomorphic sarcoma

Date	Clinical event	Findings and management
April 2025	Initial symptom onset	The patient first noted a progressively enlarging lesion on the right posterior thigh.
August 13, 2025	Primary care evaluation	Examination revealed a 4 × 2.5 cm lesion described as a “furuncle” with central fluctuance. The patient was treated with amoxicillin-clavulanate and referred for surgical evaluation.
August 22, 2025	Emergency department visit	Worsening lesion despite antibiotic therapy. Incision and drainage were performed with minimal output. The patient was discharged on cephalexin and trimethoprim-sulfamethoxazole.
September 12, 2025	General surgery evaluation	Examination demonstrated a 6 × 3 × 2 cm mass. Outside imaging suggested an exophytic lesion. The patient was referred to surgical oncology.
September 16, 2025	Surgical oncology consultation	Operative excision of the lesion was planned.
September 18, 2025	Initial surgical excision	Excision of the right posterior thigh mass was performed. Pathology demonstrated FNCLCC grade 3 UPS with positive surgical margins.
October 2025	Postoperative staging evaluation	MRI of the right lower extremity demonstrated postoperative changes without residual enhancing mass. CT of the chest, abdomen, and pelvis demonstrated no metastatic disease.
November 2025	Wide local re-excision	Re-excision with wide margins was performed. Pathology demonstrated no residual sarcoma and negative margins.
December 2025	Surveillance imaging at Memorial Sloan Kettering Cancer Center	Surveillance CT imaging identified multiple pulmonary lesions. Biopsy confirmed metastatic sarcoma involving the lungs.
December 2025 to March 2026	Systemic oncologic management	The patient enrolled in an immunotherapy-based clinical trial and continued multidisciplinary oncologic follow-up.
March 2026	Latest follow-up	Local examination of the surgical site demonstrated no evidence of local recurrence. The patient remained under active oncologic surveillance.

Postoperative imaging, including MRI of the right lower extremity (Video 1) and CT of the chest, abdomen, and pelvis, demonstrated no residual tumor or metastatic disease.

**Video 1:** Postoperative MRI of the right posterior thigh demonstrating no residual enhancing tumor MRI demonstrating post-surgical changes within the subcutaneous tissues of the right posterior thigh/buttock region, with associated edema and granulation tissue. No evidence of residual enhancing tumor or drainable fluid collection is identified.

Given the positive margins, the patient underwent wide local re-excision on November 20, 2025. The procedure included en bloc resection of the prior surgical bed with 4 cm circumferential margins and deep extension to the fascia lata. No gross residual tumor was identified intraoperatively.

## Discussion

UPS is a high-grade soft tissue sarcoma that most commonly affects older adults and is rarely seen in adolescents and young adults [1,2]. Despite its rarity in this age group, the clinical and histopathologic features remain consistent across age distributions, with no identifiable line of differentiation after comprehensive evaluation [1]. Clinically, UPS most often presents as a painless, progressively enlarging soft tissue mass, typically involving the extremities or trunk [4]. However, its presentation is frequently nonspecific, which can lead to misdiagnosis and delayed treatment. In the present case, the lesion was initially interpreted as an infectious process and managed with antibiotics and incision and drainage, without clinical improvement. Such diagnostic delays are well described in the literature, where UPS and other soft tissue sarcomas may mimic benign inflammatory or infectious conditions [6,8].

Recognition of “red flag” features is essential for early diagnosis. These include lesion size greater than 5 cm, deep fascial involvement, progressive growth, and failure to respond to appropriate medical therapy [8]. In this patient, continued enlargement despite treatment should have prompted earlier advanced imaging and biopsy. Histologically, UPS is characterized by a pleomorphic population of spindle and epithelioid cells with marked nuclear atypia, high mitotic activity, and tumor necrosis, without a definable line of differentiation [5]. Immunohistochemistry is typically negative for lineage-specific markers, reinforcing the diagnosis as one of exclusion [5]. In this case, extensive immunohistochemical workup and molecular testing, including negative MDM2 amplification, helped exclude other high-grade sarcomas such as dedifferentiated liposarcoma.

Molecular profiling studies have demonstrated that UPS is biologically heterogeneous, with distinct genomic and immune subgroups that may correlate with clinical outcomes and therapeutic sensitivity [3]. Despite this heterogeneity, complete surgical excision with negative margins remains the cornerstone of treatment [3,8]. Inadequate initial excision, often referred to as an “unplanned excision,” is associated with residual tumor in a significant proportion of re-resection specimens and may increase the risk of local recurrence [9]. Adjuvant radiotherapy is commonly recommended for high-grade, large, or deep-seated tumors, or in cases with close or positive margins, as it improves local control [8]. Systemic chemotherapy is reserved for selected high-risk or metastatic cases, although its role in localized UPS remains controversial [5].

Metastatic spread occurs primarily through hematogenous dissemination, with the lungs being the most common site of metastasis [5,7,9]. Adolescents and young adults with metastatic soft tissue sarcomas often require specialized multidisciplinary care due to the aggressive nature of disease in this population [5]. Prognosis in UPS is primarily determined by tumor grade, size, and depth, with high-grade and larger tumors associated with worse outcomes [3,9]. Early diagnosis and complete surgical resection significantly improve local control and survival outcomes.

This case emphasizes the importance of maintaining a high index of suspicion for soft tissue sarcoma in any enlarging mass, particularly those that do not respond to standard treatment, and highlights the critical role of early imaging, biopsy, and multidisciplinary management in optimizing patient outcomes. Of note, a limitation of this study is the absence of germline genetic testing in the setting of a relevant family history of breast cancer.

## Conclusions

This case underscores the importance of maintaining a high index of suspicion for malignancy in soft tissue lesions that do not respond to standard treatment for infection. We present a 21-year-old female whose lesion was initially managed as an abscess but was ultimately diagnosed as high-grade UPS. UPS can present atypically in young patients and may mimic benign conditions. Early imaging, biopsy, and multidisciplinary management are essential for accurate diagnosis and optimal outcomes.
